# Supplementary material for: Impact of Superiors’ Ethical Leadership on Subordinates’ Unethical Pro-Organizational Behavior: Mediating Effects of Followership
Source: Behav Sci (Basel). 2023 May 31;13(6):454. doi: 10.3390/bs13060454 (PMC10294972; doi:10.3390/bs13060454)
Supplement: Supplementary file 1 [file behavsci-13-00454-s001.zip › behavsci-2363076-supplementary.pdf]

Table S1. Ethical Leadership Scale.

Questionnaire	Source
a1. My superior can be trusted.<br>a2. My superior listens to what employees have to say.<br>a3. My superior discusses work ethics or values with employees.<br>a4. My superior has the best interests of employees in mind.<br>a5. My superior conducts his/her personal life in an ethical manner.<br>a6. My superior makes fair and balanced decisions.<br>a7. My superior sets an example of how to do things the right way in terms of ethics.<br>a8. My superior disciplines employees who violate ethical standards.<br>a9. My superior defines success not just by results but also by the way they are obtained.	Brown et al. (2005)

Table S2. Followership Scale.

Questionnaire	Source
b1. My work helps me fulfill some societal goal or personal dream that is important to me.<br>b2. My personal work goals align with the organization's priority goals.<br>b3. I am highly committed to and energized by my work and organization, giving them my best ideas and performance.<br>b4. My enthusiasm also spreads to and energizes my co-workers.<br>b5. Instead of waiting for or merely accepting what the superior tells me, I personally identify which organizational activities are most critical to achieving the organization's priority goals.<br>b6. I actively develop a distinctive competence in those critical activities so that I become more valuable to the superior and the organization.<br>b7. When starting a new job or assignment, I promptly build a record of successes in tasks that are important to the superior.<br>b8. My superior can give me a difficult assignment without the benefit of much supervision, knowing that I will meet my deadline with the highest-quality work and that I will "fill in the cracks" if need be.<br>b9. I take the initiative to seek out and successfully complete assignments that go above and beyond my job.<br>b10. I independently think up and champion new ideas that will contribute significantly to the superior's or the organization's goals.<br>b11. I try to solve the tough problems (technical or organizational), rather than ask the superior to do it for me.<br>b12. I help out other co-workers, making them look good, even when I do not get any credit.<br>b13. I help the superior or group see both the upside potential and downside risks of ideas or plans, playing the devil's advocate if need be.<br>b14. I understand the superior's needs, goals, and constraints, and work hard to help meet them.<br>b15. I actively and honestly own up to my strengths and weaknesses rather than put off evaluation.<br>b16. I make a habit of internally questioning the wisdom of the superior's decision rather than just doing what I am told.	Kelley (1994)

b17. I act by my own ethical standards rather than the superior's or the group's standards.	
b18. I assert my views on important issues, even though it might mean conflict with my group or reprisals from the superior.	

Table S3. Unethical Pro-Organizational Behavior (UPB) Scale.

Questionnaire	Source
c1. If it helped my organization, I would misrepresent the truth to make my organization look good.<br>c2. If it helped my organization, I would exaggerate the truth about my company's products or services to customers and clients.<br>c3. If it benefitted my organization, I would withhold negative information about my company or its products from customers and clients.<br>c4. If my organization needed me to, I would give a good recommendation on behalf of an incompetent employee who may become another organization's problem instead of my own.<br>c5. If my organization needed me to, I would withhold inadvertently misrepresenting information to relevant agencies.<br>c6. If needed, I would conceal information from the public that could be damaging to my organization.	Umphress et al. (2010); Lee and Jeon (2016)
